# Expert stakeholders on the role of qualitative research in World Health Organisation guidelines

**DOI:** 10.1093/heapol/czaf105

**Published:** 2026-02-11

**Authors:** Melissa Taylor, Paul Garner, Sandy Oliver, Nicola Desmond

**Affiliations:** Department of Clinical Sciences, Liverpool School of Tropical Medicine, Liverpool L3 5QA, United Kingdom; Department of Clinical Sciences, Liverpool School of Tropical Medicine, Liverpool L3 5QA, United Kingdom; EPPI-Centre, Social Science Research Unit, UCL Institute of Education, University College London, London WC1H 0NS, United Kingdom; Africa Centre for Evidence, Faculty of Humanities, University of Johannesburg, Johannesburg 2092, South Africa; Department of International Public Health, Liverpool School of Tropical Medicine, Liverpool L3 5QA, United Kingdom; Department of Global Health and Development, London School of Hygiene and Tropical Medicine, London WC1E 7HT, United Kingdom

**Keywords:** qualitative research, qualitative evidence synthesis, guideline development, global health

## Abstract

Qualitative research findings are sometimes used in guideline development, but usually in an ad hoc manner. We sought to explore how qualitative research could contribute to guideline development, identify examples of qualitative research being used to inform guideline development, and gather suggestions for how qualitative research might be incorporated more systematically in guideline development. Using a topic guide, in 2022–24, we interviewed experts who had participated in World Health Organization (WHO) guideline development. We used purposeful sampling, including qualitative researchers, guideline developers, guideline panel members, and implementation researchers. We interviewed 16 participants, and identified three themes: (i) respondents endorsed using qualitative research findings in developing WHO guidelines, and highlighted examples where this approach had been useful; (ii) recommendation questions in the guideline process are built on clinical decision-making, which can sometimes be too detached from social contexts for broader health problems; (iii) using qualitative research findings to help delineate context has a greater potential role in guidelines. We interpret these findings to indicate that qualitative research could be used more systematically, particularly to inform a broader framing of a health problem, or later in recommendations, to tailor to particular contexts.

Key messages

- Despite recognizing the value of qualitative research, stakeholders agreed there is still potential for more systematic use of qualitative research in WHO guideline development
- Clinical guidelines are often framed simplistically. For some questions, this may overlook the broader social context.
- One value of qualitative research is related to ‘contextual information’ but exactly how this is achieved has not been delineated.

## Introduction

The development of medical and public health guidelines is underpinned by the principles of evidence-based medicine (Sackett *et al.* 1996). This includes the use of rigorous and timely systematic reviews of research evidence (Sackett *et al.* 1996). Historically, these systematic reviews have centred around the synthesis of clinical efficacy data but have expanded in recent years to include patient and healthcare worker perspectives on relevant interventions (Lewin *et al.* 2019). One of the strengths of qualitative research is its ability to explain processes and patterns of human behaviour that can be difficult to quantify (Foley and Timonen 2015). In doing so, qualitative research can help decision-makers understand how effects observed in ideal trial conditions will translate into real-life applications (Nordon *et al.* 2016). This might include understanding: unintended consequences, perceptions of intervention benefits and harms, barriers and facilitators to programme success, the influence of personal and local context on health behaviour, and how broader systems function and their impact on people (Patton 2015, Tolley *et al.* 2016, Taylor *et al.* 2024).

**Figure 1:**
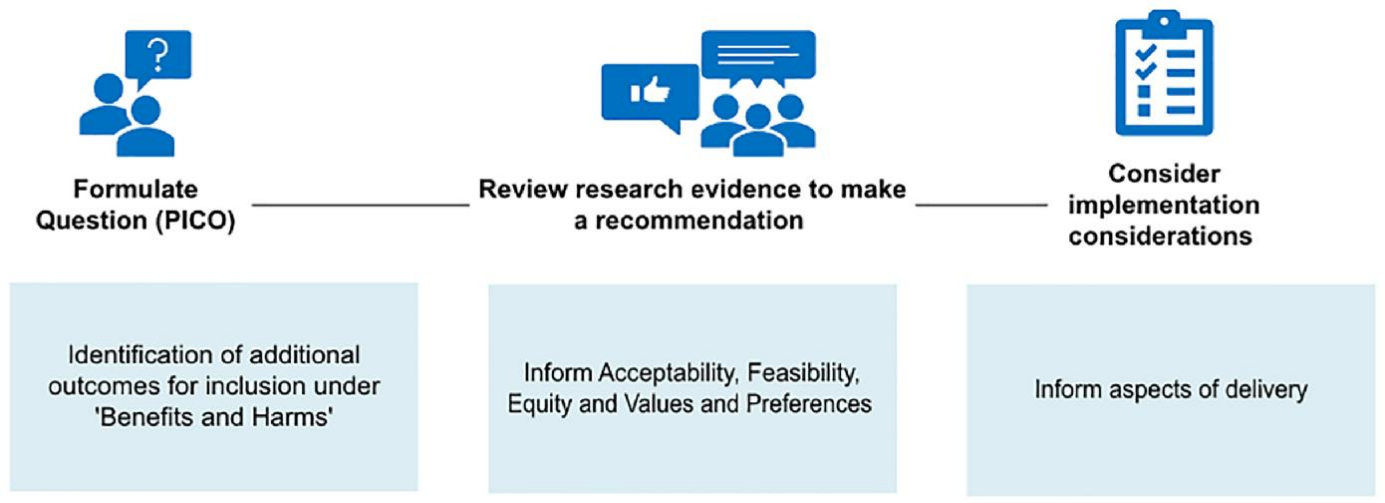
Uses of qualitative research in the three-stage GRADE evidence to decision process. PICO, Participants, Intervention, Comparator, Outcome.

One of the most frequent users of qualitative research in guideline development is the World Health Organization (WHO) (Wang *et al.* 2020). In a 2019 series of papers, authors collaborating with WHO published guidance on how qualitative research might be used in three key stages of guideline development, depicted in Fig. 1: defining the scope, formulating the recommendations and defining the implementation considerations.

Firstly, qualitative research has sometimes been used to help define the guideline scope (Downe *et al.* 2019). Typically, ‘scope’ encompasses the intervention(s) of interest, relevant comparator interventions, the target population, and the outcomes that will be evaluated (WHO 2015). Downe *et al.* (2019) highlight the 2018 WHO guideline on Intrapartum Care for a Positive Childbirth Experience, which was informed by qualitative research that led to the inclusion of a ‘positive birth experience’ and pain management outcomes.

Second, WHO are using qualitative research alongside other research evidence in evidence-to-decision frameworks (Lewin *et al.* 2019, Taylor *et al.* 2024), which were introduced to replace unstructured group evaluations prone to bias for a systematic and transparent assessment of critical criteria (Alonso-Coello *et al.* 2016). One of the most popular frameworks is the GRADE-EtD (Grading of Recommendations Assessment, Development, and Evaluation evidence to decision) (Alonso-Coello *et al.* 2016) developed in 2016, which guides the discussion around several criteria. The first includes anticipated effects; then, because an intervention can sometimes affect several competing effects, the panel consider comparative priorities termed ‘values and preferences’; they then consider resource use; how the recommendation may impact on population equity; whether the new intervention is acceptable to the population and whether it is feasible to implement. World Health Organization sometimes commission systematic reviews, particularly of qualitative research, to help inform the discussions around acceptability, feasibility, values and preferences, and equity (Alonso-Coello *et al*. 2016). For example, Lewin *et al.* (2019) highlight the forthcoming WHO ‘guidance on communication interventions to inform and educate caregivers on routine childhood vaccination in the African Region’. This guideline incorporated evidence that population groups with low levels of trust in the government may struggle to trust vaccination information delivered by government health workers in the guideline discussions around equity. Alternative frameworks, such as WHO-INTEGRATE (Rehfuess *et al.* 2019), have been developed in recent years to inform complex decision-making more effectively and include additional human rights components, socio-economic acceptability, and other considerations which may benefit from qualitative research, but have had limited traction.

A third area is the implementation considerations (Glenton *et al.* 2019). Guidelines may produce implementation considerations to guide the adaptation and implementation of an intervention at national or sub-national level (WHO 2015). Glenton *et al.* (2019) highlight the WHO guideline on digital interventions for health system strengthening which considered strategies such as allowing users to unsubscribe or determine the frequency of communication content as a result of qualitative research identifying concerns about communicating health information on mobile devices due to the risk of confidentiality breaches on sensitive topics.

An important methodological advance in helping integration has been the establishment of rigorous methods for qualitative synthesis (Noyes *et al.* 2018). There are now over 30 methods for qualitative synthesis, including framework synthesis, thematic synthesis, and meta-ethnography, which are frequently used in guidelines (Booth *et al.* 2016). Another development is GRADE-CERQual (Confidence in the Evidence from Reviews of Qualitative research), which provides guidance on assessing how much confidence to place in the findings of a qualitative synthesis, thereby aiding in decision-making in a manner similar to long-established GRADE approaches for quantitative systematic reviews (Lewin *et al.* 2018).

Nevertheless, some research has suggested that qualitative research may be used unsystematically across different guidelines (Taylor *et al.* 2024). A recent document analysis examined guidelines published between 2018 and 2020 and found that while qualitative research informed 100% of guidelines in maternal health, it informed only 20% of guidelines in infectious diseases between 2018 and 2020. Use of qualitative research in other guideline organizations, such as UK’s National Institute for Clinical Effectiveness may be lower still, informing 27% of all guidelines between 2015 and 2019 (Carmona *et al.* 2022). When qualitative research does inform guidelines, it is primarily used to inform the guideline recommendations but rarely the guideline scope or implementation considerations (Taylor *et al*. 2024). Wang *et al.* (2020) similarly reported that 86% of WHO and UK, USA and Canadian national guidelines used qualitative research to inform recommendations, but only 20% to identify scope and 19% to inform implementation considerations (Wang *et al.* 2020).

This study aims to explore how qualitative research could contribute to guideline development, identify examples of qualitative research being used to inform guideline development, and gather suggestions for how qualitative research might be incorporated more systematically in guideline development.

## Methods

### Study design

We conducted a qualitative study employing semi-structured deliberative interviews with key stakeholders. This study is based on grounded theory, which is concerned with the generation of research theory outside of the authors’ preconceptions and favours an iterative approach to collection and analysis (Noble and Mitchell 2016). Multiple aspects of the study methods were informed by a grounded theory approach, including participant recruitment, timing of data collection, and methods of analysis, which are detailed below.

### Recruitment and sample selection

We used purposive sampling to identify qualitative researchers, guideline developers, guideline panel members, and implementation researchers. The sample was chosen to represent those with direct experience of producing and or using WHO guidelines, as well as those with in-depth experience of qualitative methodology. Participants were initially identified from the authors’ existing networks and published literature. The primary investigator contacted individuals via email to identify those willing to be involved and used a snowball sampling approach to identify further contacts. Recruitment continued until data saturation was achieved, as established by consensus at author group meetings. In line with a grounded theory approach, recruitment and analysis occurred simultaneously; this meant that findings that emerged during analysis sometimes led to the decision to recruit further participants that could offer further insight. We provide an overview of the participants who agreed to participate, including their sex, professional affiliation, and country where they are currently based (Table 1).

**Table 1:** Participant demographics.

Participant demographic	Number
**Professional affiliation**	
Qualitative researcher	9
Guideline Development Group (GDG) member	2
Guideline developer	1
Guideline methodologist	2
WHO consultant	2
**Sex**	
Male	8
Female	8
**Years of experience**	
0–5	2
6–10	5
10+	9
**Country of residence**	
UK	7
Norway	2
Sweden	1
Switzerland	1
France	1
USA	1
South Africa	1
Malawi	1
Australia	1

### Data collection

The first author conducted the interviews online via Microsoft Teams during 2022–24. The typical duration of an interview was between 30 and 60 min. A topic guide was used to guide interviews. This was piloted with the first two interviews and further refined using an iterative approach. In line with grounded theory methods, data collection and analysis occurred simultaneously, meaning that the interview guide was revised when new findings emerged that required further exploration. This work is one component of the first authors PhD thesis, which was explained to participants on recruitment and at the start of the interview. Ethical review and approval were not considered necessary by the host institution as the study collected professional opinions only. Regardless, we followed standard ethical procedures, which included obtaining informed written consent, providing participant information sheets, and obtaining verbal confirmation of rights at the start of the interview. All participants read and agreed to the final publication.

Initially, we asked participants open-ended questions. However, where appropriate, we chose to follow-up on their response using a deliberative approach whereby the interviewer may provoke responses by indicating or suggesting alternate narratives and positions (Berner-Rodoreda *et al.* 2020). This method aims to construct knowledge by means of challenging and being challenged by participants and is particularly suited for interviews with senior experts and professionals as they are more likely to be comfortable with the approach (Berner-Rodoreda *et al.* 2020).

We also asked participants to provide examples of where qualitative research had informed guideline development and to explain why they felt it was an effective example. We extracted the guideline document and associated qualitative study and recorded our assessment, source, participant rationale, and our judgement in Table 2.

**Table 2:** Examples of where qualitative research has informed guideline development, recommended by participants.

Guideline	Qualitative evidence used?	Why noteworthy?	Comment
Home health records for maternal and child health (World Health Organisation 2018)	Yes: qualitative evidence synthesis	The synthesis contributed to the decision: the quantitative evidence was limited	There were no trials of the benefits of mothers holding their own records. The qualitative studies showed how much mothers liked being able to do this.
Pre-exposure prophylaxis for HIV injection drug users (World Health organisation 2016)	Yes: single study	The study overrode a guideline decision: it highlighted substantial unforeseen consequences	The trial data favoured the intervention. The qualitative research suggested that the intervention would discourage governments from funding other proven interventions to prevent Human Immunodeficiency Virus (HIV).
Tobacco: preventing uptake, promoting quitting, and treating dependence (NICE 2021)	Yes: single study	The study helped to show why an intervention was unlikely to work	Clear example of where qualitative research helped to understand the problem from the patient perspective—highlighting implementation needs in the process.
Maternal and newborn care for a positive birth experience (World Health Organisation 2022)	Yes: qualitative evidence synthesis	The synthesis contributed to the decision	Extensive analysis commissioned to inform feasibility, equity, acceptability and values and preference decisions. However, the link between the research findings and the recommendations made is unclear.
Digital interventions for health system strengthening (World Health Organisation 2019)	Yes: qualitative evidence synthesis	The synthesis influenced implementation considerations	Detailed analysis of implementation considerations organized according to leadership and government, strategy and investment, legislation policy and compliance, services and applications, infrastructure, standards and interoperability, workforce, human rights and equity. However, several findings that may have been useful to implementation were not included.

We used the in-built transcription feature on Microsoft teams before reading and editing the transcripts by hand. We ensured anonymity and confidentiality of participants by assigning unique number codes to the transcripts and quotations subsequently presented in the results.

### Data analysis

We used thematic analysis as described by Braun and Clarke (2021). The first author analysed the qualitative data with ATLAS.ti software. She coded inductively and developed a coding framework to apply to subsequent transcripts. In line with a grounded theory approach, this was an iterative process whereby the framework was consistently revised and updated as findings emerged. We retained accounts that differed from the emerging understanding of the situation as divergent codes. At this stage, the first author met regularly with the wider author team to reflect on emerging themes as a group. Here, authors interpreted the meaning behind the data and thought about the relationships between codes, themes, and hierarchies of themes. After the final report was drafted, we approached interviewees for feedback on the themes and to ensure their perspectives were adequately represented.

## Results

Overall, 50 experts were approached for an interview, and 16 agreed to participate. Those that were interviewed consisted of nine qualitative researchers, of whom five had experience in qualitative synthesis. Two participants had experience as a guideline development group member for topics in infectious disease, malnutrition and maternal health. Both of these members are clinicians by background. We further interviewed two guideline methodologists and one guideline developer. Finally, we interviewed two WHO programme consultants with experience of implementing guidelines, although not developing them. All of those interviewed had experience using or producing qualitative evidence, all qualitative researchers had experience of producing evidence for guidelines and/or attending guideline meetings. An overview of participant demographics can be found in Table 1. Those who declined to participate included 18/26 (69%) of qualitative researchers, 9/11 (82%) guideline developers, and 8/13 (62%) guideline panel members. The majority (31/34; 91%) did not respond to the invitation, and 3/34 (9%) were unavailable.

### Theme 1: Respondents endorsed using qualitative research findings in guideline development with a few illustrative examples of effective use

Respondents endorsed that qualitative research, referring to either synthesis or primary research, should be used in guideline development. We asked participants to provide strong examples of how qualitative research had impacted guideline development, the details of which are presented in Table 2. Some guideline examples provided by clinicians had collated the opinions of panel members or referenced programme evaluation studies rather than qualitative research. This observation aligns with a recurring perception among the participants that panel members are often inexperienced in qualitative methods, which limits their ability to engage with this form of evidence.

A lot of people can't really comment that well [on the qualitative research], so it wasn’t really interrogated or discussed that much. It was just sort of okay, move on. Whereas all the lively discussions were like really technical about statistics and clinical trials and stuff. I think mostly because that's what people care about and are experts in as well. (P12; GDG member, clinician)

While this view may suggest that further training in qualitative methods may be necessary for those involved in guideline development, conflicting participant views argued that this may not be a feasible expectation, given the quick turnaround of guidelines and the fact that busy clinicians volunteer their time. Our suggestion to train guideline methodologists, on the other hand, was supported by participants.

Most examples were of cases where qualitative research informed the recommendation. In one example (WHO 2018), the quantitative evidence was sparse and of low certainty. Qualitative research informed the values and preferences underlying the recommendation, suggesting that the intervention was desirable to communities, which led to a recommendation for the intervention despite its unclear efficacy. The second example demonstrated how qualitative research had overturned a recommendation, as it had identified unforeseen consequences, despite quantitative data demonstrating efficacy and safety (WHO 2016). The third example demonstrated how qualitative research explained the observed heterogeneity in quantitative effectiveness data. As a result, panel members were able to recommend the intervention alongside implementation considerations that addressed the community concerns (NICE 2021).

‪‪That's all of the additional contextual information that we gleaned through it. So we knew at the start of it that people from most socioeconomically deprived circumstances were much more likely to smoke in pregnancy. But we found out why, like actually the coping mechanism that it was part of just the socialisation actually … So it wasn't that the intervention didn't work, it was never the intention to quit (P11, qualitative researcher).

Two participants cited the WHO recommendations on maternal and newborn care for a positive postnatal experience (WHO 2022) as a strong example of qualitative research informing a guideline. Here, the guidelines incorporated extensive and detailed qualitative findings under the aspects of feasibility, acceptability, equity, values, and preference within the evidence-to-decision framework. This example is already featured in early guidance for using qualitative research in guideline processes (Lewin *et al.* 2019).

Finally, one participant provided an example of qualitative research informing detailed implementation considerations that covered a range of health system components such as policy, services, infrastructure and human rights (WHO 2019). However, one qualitative researcher felt that there is a lack of strong examples of qualitative research informing guideline development, explaining that if such examples existed, they would be well known.‪

### Theme 2: The questions a guideline asks are often based on clinical decision-making, which can sometimes be too finely focused for broader health problems

#### The questions a guideline asks are often a choice between narrow clinical or technical options, which may not benefit from qualitative research

Several participants acknowledged that whether qualitative research is helpful depends on the guideline question, including the intervention of interest. One participant further argued that the value of qualitative research is limited for narrow clinical interventions, such as a choice between two drugs or drug regimens. However, participants who also had experience of delivering policies or care to communities felt that guidelines are sometimes overly focused on narrow clinical or technical interventions that might overlook the underlying community concerns. For example, one participant expressed frustration at the lack of engagement with more fundamental questions that are sociopolitical in nature, arising from communities in her research area.

I got there [the guideline panel] and I realized…It was about how to increase the amount of [the intervention drug] to make it more regular….Umm, which was a bit kind of disheartening, In terms of the lack of engagement with the real issues and recognizing that there was some quite profound challenges with proceeding in its current format. (P05, qualitative researcher)

Another participant explained that guideline questions often start with a pre-determined technical solution, rather than understanding the health problem itself.

In most of our guidelines now, it's not how to solve the problem, it’s ‘here's the solution that we decided on from Geneva’. Before DDT [insecticide], malariologists were trained as problem solvers. After DDT, malariologists were trained as solution implementers. (P14, implementation researcher, referencing José Najera, former WHO epidemiologist (Nájera *et al.* 2011)

One might argue that an emphasis on technical interventions may inadvertently limit autonomy at national levels. For example, two participants gave examples of how the top-down process of WHO guidance can inadvertently disempower local actors to address health problems. In the case of malaria prevention, endorsement of one specific intervention by WHO may lead to poor adherence where another intervention was more practical or socially desirable. In schistosomiasis, guidelines for implementing mass drug administration may limit financial and political investment in other approaches.

‪So you know, in Uganda more for example, it seemed absolutely bizarre to me that on the one hand, they're putting pouring money into distributing praziquantel to fishermen, but on the other hand, they had already in their regulations a whole host of things which required people to have pit latrines or access to water pumps and stuff like that. And they pay no attention to that at all and never enforced it and had no interest in enforcing it. (P05, qualitative researcher)

#### There is an opportunity to use qualitative findings to help frame the questions a guideline asks

While participants expressed concern that the clinical or technical focus of guidelines could overlook significant variations in patient behaviour and broader systemic issues, qualitative researchers argued that many of these issues can only be understood through qualitative research.

If we know that that pill A is vastly clinically effective and yet we're not seeing clients using that pill and getting better and they're then obviously there's something else going on there and that's something is probably behavioural, it's probably cultural, it's probably social and that sort of stuff is another thing that qualitative research can help to unpick. (P04, qualitative researcher)

This quote seems to suggest that a ‘problem solving’ perspective informed by qualitative research might broaden the potential solutions a guideline explores. Comments regarding the framing of guidelines were raised by several other participants. Some participants felt that qualitative research could help to inform what questions a guideline might ask at the outset, by understanding what patients and health professionals understand about the ‘issues’ of importance.

So I guess if you were wanting to use kind of a higher theoretical frame, I think that can help kind of formulate the thinking around where a guideline might need to go before you start. (P11, qualitative researcher)

A qualitative researcher gave a potential example of a reframed guideline question: ‘Well, actually, you don't always want to think of it in terms of a specific health problem. You might actually want to think of it as something more about how to live healthily. What's the physical activity that helps? What's the healthy eating that helps, you know?’ (P09, qualitative researcher).

Some participants argued that health guidelines should extend beyond technical interventions and take a health systems approach, which may require comprehensive, and contextually tailored approaches rather than a one-size-fits-all solution. One participant gave an example of tuberculosis (TB) rapid diagnostics, where qualitative research had demonstrated fundamental and complex requirements for success.

‪Well, I guess there should be recommendations for health systems, not just recommendations for health practice or laboratory practice or you know technical things. You can't assume that every health system is going to be the same, but maybe when you've got things that absolutely have to adapt to their particular context, it's the understanding that is more important than the point estimate. (P09, qualitative researcher)

### Theme 3: Using qualitative research findings to help delineate context has a greater potential role in guidelines

‪‪‪The idea of ‘context’ was frequently referenced as the key value of qualitative research by both those with experience implementing recommendations and qualitative researchers. They further argued that the loss of contextual information can render guidelines unfit for purpose, as it becomes unclear within which settings the recommendations are appropriate or how to implement them under specific conditions.

You know, there's a tendency to in the search for collaboration and consensus to take the kind of the local or the contextual, or for the political out of the equation and just to focus on the technical aspects. And in so doing, you actually end up making those guidelines not fit for purpose. Because they can't be applied routinely in all contexts across multiple continents.. (P05, qualitative researcher)

Guideline developers, on the other hand, viewed this loss of context as necessary, as global guidelines need to be generalizable. They further argued that contextualization was instead something expected of national and local guideline developers when adapting WHO guidelines.

‪You want to be concerned to ensure that the views put forward for the guideline panel to interpret… are as generalizable as possible. I think generalizability of the outcomes is the number one concern. (P07, Guideline developer)

On the other hand, a qualitative researcher argued that qualitative findings can be shared across multiple populations based on shared contexts and underlying causes, and this might also extend to high-income countries and low- and middle-income countries. The participant here seems to be referencing ‘transferability’, which describes the application of qualitative findings drawn from one population to another that has not been directly studied (Carminati 2018).

‪I wouldn't say countries and I really dislike the LMIC/HIC label. So, I always say some things like, in contexts where access to power is limited, charging a cell phone maybe difficult…There's parts of Canada that are very poor, or have very limited access to power, you know, so we're trying to sort of highlight contextual factors that could be anywhere in the world. (P06, qualitative researcher).

Other qualitative researchers echoed this point and argued that findings from qualitative syntheses have greater potential to be generalizable, considering they are informed by multiple settings and a large cumulative participant number.

Participants sometimes referenced theory or explanatory data as an important contribution of qualitative research but argued that current guideline processes can make it difficult to develop this data sufficiently. It could be argued that explanation and theory is necessary for the development of transferable findings relevant to multiple populations in global guidelines.

For example, one participant with experience producing qualitative evidence syntheses commissioned by WHO felt that short time frames limited their ability to undertake a detailed analysis of the data. They felt that qualitative research benefits from time for reflection, trying different perspectives, and seeking input from others; short time frames might result in missing out on higher-level conceptual and theoretical findings. A guideline developer added that when time and resources are limited, qualitative research may not be used at all; instead, aspects of acceptability, feasibility, values, preferences, and equity are addressed through quantitative surveys.

‪‘But you lose, especially with qualitative research, that time to sort of digest things, try things a different way, look at it from a different perspective, bring somebody else in to talk about it. You know, you lose the thinking time. (P06, qualitative researcher)

Beyond the time demands of guideline production, qualitative researchers also spoke of the Evidence-to-Decision framework typically used by the WHO to guide decision-making. Several participants felt that the framework encourages deconstruction and repetition of qualitative findings across multiple boxes of acceptability, feasibility, equity, and values and preferences, thereby reducing the explanatory power. Although another qualitative researcher suggested that sometimes, developing explanatory findings are limited by the primary qualitative studies included in the synthesis

‪‪The GRADE framework disaggregates the evidence, so it takes all the strength away. Some of the compelling characteristics of the evidence is in the strength of explanation, but if you fit it in, if you break it down into tiny boxes, all the links are lost, all the causal explanations are lost. (P09, qualitative researcher)

## Discussion

We set out to explore how qualitative research could contribute to guideline development, identify examples of qualitative research being used to inform guideline development, and gather suggestions for how qualitative research might be incorporated more systematically in guideline development. We found that stakeholders wanted to see qualitative evidence used in decision-making and identified several examples of early adoption. However, the narrow focus of many clinical and public health guidelines might overlook important aspects of the social context examined by qualitative research. Developing transferable or theoretically transferable findings can enhance the relevance of qualitative research in global guidance and facilitate the development of recommendations that encourage tailoring to diverse contexts. However, how this might be achieved is not yet understood.

The examples we collected of qualitative research that had informed guidelines were mostly used to inform the second stage of guideline development, formulating the recommendations. This observation aligns with studies by Taylor *et al.* (2024) and Wang *et al*. (2020) that report qualitative research rarely informs the guideline scope or implementation considerations. While it might be argued that this is because qualitative research has limited utility in these steps of guideline development, our findings suggest that there may be unmet potential in these areas.

Firstly, our findings suggest that qualitative research can be used to understand overlooked social and structural explanations for a given health problem. This might influence the questions a guideline chooses to address, including the interventions under consideration. Such an approach might be useful for guidance for complex clinical and public health interventions, especially in areas where efficacious treatment has long been established but not yet yielded intended results (Greenhalgh 2012). Previous guidelines have touched on this approach by using qualitative research to inform additional efficacy outcomes (Downe *et al.* 2019), but fall short of an in-depth bottom-up re-thinking of a health problem. Further research is needed to understand how reframing health problems may impact guideline questions and how this will, in turn, alter recommendations.

Secondly, we found that a key value of qualitative research is the ability to understand context. Our findings indicate that contextual findings applicable to multiple populations, known as transferable findings, may be particularly useful in global guideline development. We argue that greater inclusion of transferable qualitative findings may help improve the usability of guidelines by offering tailored recommendations and implementation advice. Previous research has noted the tendency for WHO to recycle entire implementation sections for multiple guidelines (Wang *et al.* 2016) and treat guideline adaptation and implementation as an afterthought (Norris and Ford 2017). However, using context-dependent findings to inform implementation is supported by the widespread use of qualitative evidence in implementation research (Damschroder 2009, Pfadenhauer *et al.* 2017, Hamilton and Finley 2019, Nilsen and Bernhardsson 2019, Smith *et al.* 2020).

Moving from a universalist guideline perspective to a one where tailoring is encouraged will impact how ‘transferable’ qualitative research is valued and used but represents a key epistemological challenge within global health (Carminati 2018, Munthe-Kaas *et al.* 2020). Developing recommendations that can be tailored requires explanatory qualitative findings with thick descriptions of meaning, interpretation, context, and relationships, all of which help to understand theoretical generalizability and transferability (Younas *et al.* 2023). Qualitative evidence syntheses are well-suited to develop theoretically transferable findings due to their ability to compare and contrast phenomena across multiple populations and contexts (Booth *et al.* 2019). Further research is required to understand exactly how best to assess and present contextualized recommendations that are useful and feasible within the remit of global guidelines. Work to date has centred around adapting guidelines for local contexts after global guideline processes (Schünemann *et al.* 2017). The Context and Implementation of Complex Interventions framework may offer an appropriate place to start, which outlines a comprehensive conceptualization of context, including geographical, epidemiological, socio-cultural, socio-economic, ethical, legal, and political (Pfadenhauer *et al.* 2017).

### Strengths and limitations

This study drew on a diverse range of experts, many of whom had extensive experience in the field. The author team itself represents a variation in experience of guideline methodology, social sciences, and qualitative synthesis, which allowed for a thorough analysis of results. However, there remains an underrepresentation of guideline developers in the sample. Although efforts were made to recruit participants from this group, most were unable to participate. This may reflect the busy nature of guideline developers, a discomfort disclosing their perspectives, or a lack of interest and priority given to qualitative research. As a result, the perspectives in this study are skewed towards those of qualitative researchers. Those interviewed predominantly spoke from their experience of clinical guidelines. We captured one example of where a participant had worked on health systems guidance and noted that this was where qualitative research was particularly helpful for developing implementation considerations.

## Conclusion

Qualitative research can contribute to better guidance; however, its use is sometimes inconsistent and unsystematic. Further potential uses of qualitative research include informing a broader conceptualization of a health problem and developing recommendations that encourage tailoring to different contexts. Further research should bring together a range of experts, including guideline developers, to build on the findings and explore additional methods for incorporating qualitative research into guideline development.

## Data Availability

The data underlying this article will be shared on reasonable request to the corresponding author.

## Reflexivity statement

The first author is a female and early career researcher, and the study was completed as part of her PhD studies. Her thesis explores the current and future application of qualitative research to guideline development. She has experience in conducting several Cochrane qualitative and quantitative systematic reviews on topics of Global Health. Participants were recruited from the author team’s personal networks, and the first author knew two of the participants personally.

PG is a clinical epidemiologist who established the Cochrane Infectious Diseases Group and was instrumental in developing its portfolio over 30 years, as well as contributing to the development of evidence to decision-making procedures. He has participated in qualitative evidence synthesis and believes that it could have a more important role in making guidelines better, but that WHO's culture is predominantly medical and quantitative, so change requires more substantive restructuring of the process.

SO is a social scientist bringing extensive experience of synthesizing quantitative and qualitative studies. She has acted as a methodologist for a WHO guideline panel and has a special interest in combining and building on the knowledge of policymakers, practitioners and communities to explore health and social problems to make collective decisions.

ND is a professor of anthropology and global health with extensive experience in empirical, qualitative research in LMIC and working within multi-disciplinary teams in implementation research and public health interventions. She has participated in meta-ethnographic studies and in WHO technical working groups. She has contributed to driving the recognition of interpretive epistemology within the global health agenda and to building capacity for rigorous qualitative contributions to global public health problems.
